# Monte Carlo Calculation of Radioimmunotherapy with ^90^Y-, ^177^Lu-, ^131^I-, ^124^I-, and ^188^Re-Nanoobjects: Choice of the Best Radionuclide for Solid Tumour Treatment by Using TCP and NTCP Concepts

**DOI:** 10.1155/2015/284360

**Published:** 2015-06-02

**Authors:** S. Lucas, O. Feron, B. Gallez, B. Masereel, C. Michiels, T. Vander Borght

**Affiliations:** ^1^Research Centre for the Physics of Matter and Radiation (PMR), University of Namur, 61 Rue de Bruxelles, 5000 Namur, Belgium; ^2^NAmur Research Institute for LIfe Sciences (NARILIS), University of Namur, 61 Rue de Bruxelles, 5000 Namur, Belgium; ^3^Pharmacology and Therapeutics Unit (FATH), Institute of Experimental and Clinical Research (IREC), Université Catholique de Louvain (UCL), 53 Avenue Mounier, 1200 Brussels, Belgium; ^4^Biomedical Magnetic Resonance Group (REMA), Louvain Drug Research Institute, Université Catholique de Louvain (UCL), 73 Avenue Mounier, 1200 Brussels, Belgium; ^5^Namur Medicine and Drug Innovation Center (NAMEDIC), University of Namur, 61 Rue de Bruxelles, 5000 Namur, Belgium; ^6^Unité de Recherche en Biologie Cellulaire (URBC), University of Namur, 61 Rue de Bruxelles, 5000 Namur, Belgium; ^7^Centre for Molecular Imaging, Radiotherapy and Oncology (MIRO), Institute of Experimental and Clinical Research (IREC), Université Catholique de Louvain (UCL), 1 Dr. G. Therasse, 5530 Yvoir, Belgium

## Abstract

Radioimmunotherapy has shown that the use of monoclonal antibodies combined with a radioisotope like ^131^I or ^90^Y still remains ineffective for solid and radioresistant tumour treatment. Previous simulations have revealed that an increase in the number of ^90^Y labelled to each antibody or nanoobject could be a solution to improve treatment output. It now seems important to assess the treatment output and toxicity when radionuclides such as ^90^Y, ^177^Lu, ^131^I, ^124^I, and ^188^Re are used. Tumour control probability (TCP) and normal tissue complication probability (NTCP) curves versus the number of radionuclides per nanoobject were computed with MCNPX to evaluate treatment efficacy for solid tumours and to predict the incidence of surrounding side effects. Analyses were carried out for two solid tumour sizes of 0.5 and 1.0 cm radius and for nanoobject (i.e., a radiolabelled antibody) distributed uniformly or nonuniformly throughout a solid tumour (e.g., Non-small-cell-lung cancer (NSCLC)). ^90^Y and ^188^Re are the best candidates for solid tumour treatment when only one radionuclide is coupled to one carrier. Furthermore, regardless of the radionuclide properties, high values of TCP can be reached without toxicity if the number of radionuclides per nanoobject increases.

## 1. Introduction

Radioimmunotherapy uses radionuclides labelling of monoclonal antibodies (mAbs) to deliver ionizing radiation to tumour cells. Efficacy and toxicity of the treatment are mainly influenced by the antibodies biokinetics and biodistribution but also by radionuclides physical properties. Today, various *β*-emitters coupled to mAbs are being tested and compared to eradicate tumour tissues. If this technique is well adapted to treat radiosensitive hematopoietic malignancies, clinical studies have always shown poor therapeutic effects on solid and radioresistant tumours. Almost no clinical trials on patients with colorectal, ovarian, gastric, pancreatic, prostate, and breast cancer went beyond phase II [1]. This lack of success might be partly explained by the fact that these solid tumours are less sensitive to ionizing radiation and require doses larger than 60 Gy to be sterilised [2]. Today, numerous research studies are focused on optimizing radioimmunotherapy by increasing the absorbed doses through a better accumulation or a better penetration of radiolabelled antibodies. But doses still remain insufficient to trigger a good treatment response [3, 4].

Another solution to maximize the deposited doses inside the solid tumour would be to increase the number of radionuclides coupled to each antibody. Today, the coupling is performed either by covalently binding the radionuclide directly to the antibody or by crosslinking through a chelating agent or chemical linker. These labelling procedures often lead to a variable amount of radionuclides per antibody and a too large number of radionuclides per mAb may impair the antibodies immunoreactivity, leading to lower tumour accumulation and higher liver uptake [5, 6].

To preserve immunoreactivity, one could imagine to synthesize a nanoobject (NO) made of a nanoparticle (NP) containing a large number of radionuclides, which is coupled to each antibody. Indeed, it is now possible to assemble nonradioactive and radioactive atoms to form radioactive nanoparticles (NPs) [7–9]. In order to prevent their aggregation and to maintain their stability within a biological environment, the surface of these radioactive NPs can be coated with polymeric macromolecules such as plasma polymerized allylamine (PPAA), polyethylene glycol (PEG), or polysaccharide glycoprotein (Arabic gum) [10, 11]. These types of macromolecules can also prevent radioactive atoms from leaching. Moreover, NPs offer a large surface capable of accommodating more than one antibody per particle, making enhancement of uptake of these NPs inside or around the tumour possible [11]. Another solution would be to synthesize another NO made of a biocompatible nanoparticle coupled to more than one radiolabelled antibody. Experiments using this kind of nanoobjects have recently shown that they could be used for efficient cancer treatment. Indeed, in vivo imaging on animals has proved that they are preferentially distributed in the tumour mass after injection and that they present an in vivo pharmacokinetic profile very similar to that of uncoupled antibodies [12, 13].

In a first paper [14], absorbed doses (*D*) deposited by NOs made of antibodies coupled with nanoparticles containing several *β*-emitters ^90^Y were simulated by using the Monte Carlo particle transport code MCNPX (Monte Carlo N-Particle eXtended). Dose values were calculated according to a new model of spherical solid tumors in which the antibodies could be distributed uniformly, linearly, or exponentially inside the tumor volume. Preliminary results showed that viable tumor cells received absorbed doses larger than 50 Gy everywhere inside the tumor. In a second paper, the good therapeutic efficacy of these ^90^Y-NPs against non-small-cell lung cancer (NSCLC) was theoretically confirmed by determining the biological effective dose (BED) and tumor control probability (TCP) [15]. NSCLC is a good example of fast growing and radioresistant carcinoma with important hypoxic areas. BED and TCP values took into account the radiosensitivity of the targeted tissues, the doubling time of the cancer cells, the repair process of sublethal damage taking place between two irradiations, and the effect of hypoxia for which the radiosensitivity changes according to whether cells are anoxic or well-oxygenated. Early stage NSCLC is usually treated surgically, although stereotactic body radiation therapy (SBRT) may represent a good alternative when inoperable [16–19]. Unfortunately, this fast growing tumour is often diagnosed when already disseminated.

The main objective of this paper is to model the therapeutic efficacy and the complication risks of the surrounding tissue when the NOs are filled with other *β*-emitters targeting tumour cells. The assumption is that the NOs are well targeting the metastases, but the energy deposition may be influenced by the distribution inside the tumour deposits as well as the NOs radioisotope concentration.

The first part of this paper will compare dose profiles for five different *β*-emitters (^90^Y, ^188^Re, ^177^Lu, ^124^I, and ^131^I) to see which one is the most efficient for treatment when only one radionuclide is coupled to each antibody. The second part is to establish, for the five different *β*-emitters, the relationship between TCP and NTCP when the number of radioactive atoms per NO varies. TCP and NTCP curves were analyzed for three types of NO distributions (uniform, linear, and exponential), with a tumoral biological half-life of 6 days and for two tumour sizes (0.5 cm and 1.0 cm radii). For each TCP curve, the lowest number of radioactive atoms per NO required to obtain a maximum TCP of 100% will be determined, as well as the total doses deposited inside the tumours and deposited in the surrounding tissues.

It has to be noted that it is not the intention of this paper either to determine the amount of activity to administer to a patient or to establish a treatment planning (for that purpose, please see the review given by [20]). Instead, one has to consider our results as advised to researchers who want to develop NO-based radioimmunotherapy.

## 2. Material and Methods

**Figure 1:**
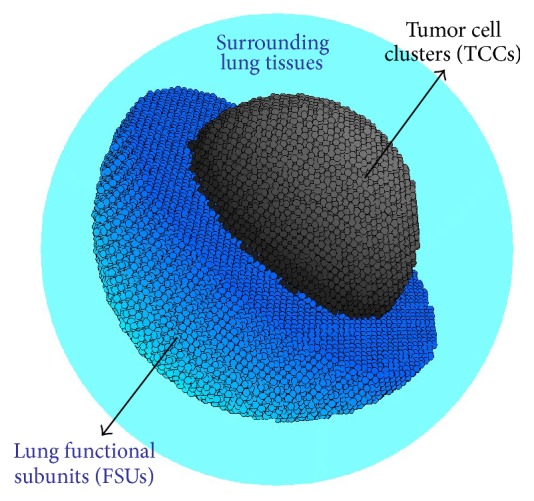
Three-dimensional representation of a NSCLC tumour surrounded by lung shell. The tumour sphere is subdivided into 250 *μ*m diameter spherical tumour cell clusters (TCCs) and the lung shell is subdivided into 250 *μ*m diameter functional subunits (FSUs).

For our simulations with the MCNPX software, the NSCLC tumour is represented by a sphere subdivided into a set of 250 *μ*m radius spherical tumour cell clusters (TCCs) arranged in a simple cubic lattice structure as represented in Figure 1 [15]. The surrounding lung tissue is described by a 4.0 cm thick spherical shell also subdivided into small 250 *μ*m radius spherical functional subunits (FSUs) also arranged in a cubic lattice. The composition and density of each cell unit are taken from ICRU-44 soft tissue for the TCCs and from ICRU-44 lung tissue for the FSUs.

For our geometric model, we also assume that the FSU and TCC are surrounded by blood vessels which could be either a new blood network created through angiogenesis or simply the preexisting vasculature. Consequently the radiolabelled antibodies or the radioactive nanoobjects can penetrate within the lung tissues or the tumour and surround the different cell units. So, the surface of each TCC and each FSU becomes a potential source of radiation characterized by a probability of emission. The latter will be similar for all FSUs, meaning that the distribution of the NO in the spherical shell is uniform. Inversely, for the tumour, the probability of emission can be similar for all TCCs or can decrease linearly and exponentially from the surface towards the tumour centre. Finally, if the targeting function of the antibodies is preserved, the TCC emission probability should be higher than the FSU emission probability. According to the experimental results given by Wiseman et al. [21], for patients with non-Hodgkin's lymphoma (NHL) treated with ^90^Y Zevalin, the mean tumour-to-lung deposited dose ratio corresponded to 7, meaning that the doses absorbed by the tumours are seven times higher than the doses deposited in lung tissues. The mean tumour volume value was 42.5 cm³. So, for our simulations, we have assumed that the TCC emission probability is seven times more important than the lung FSU emission probability.

In this work, the treatment of small NSCLC tumours by RIT will be investigated for five different radionuclides according to MCNPX simulations: one pure *β*-emitter (^90^Y), two *β*-emitters with a low-abundance *γ* (^177^Lu and ^188^Re), and two *β*-emitters with a higher *γ* abundance (^131^I and ^124^I). ^131^I is the most extensively used radionuclide in RIT because of its availability, its ease for chemical conjugation, and its ability to perform *γ* imaging and *β* therapeutic studies with the same biological vector. ^131^I- and ^90^Y-labelled mAbs are mainly used to treat patients with NHL. However, numerous phase I-II clinical trials have been reported for patients with solid tumours [22]. Despite the high-energy *β*-particles emitted by the ^188^Re, the clinical trials done with this radionuclide only concern the treatment of patients with haematological malignancies [23, 24]. Inversely, the efficacy of the low-energy *β*-emitter ^177^Lu was only assessed to treat solid tumours such as ovarian or prostate cancer [25–27]. More recently, numerous research studies into ^124^I have been investigated because this high-energy positron-emitting radionuclide is suitable for PET imaging and radioiodine therapy [28, 29]. The main physical properties of these five radionuclides are detailed in Table 1. *T*_1/2_^phys^ is the physical half-life of the five different radionuclides. *p*_*γ*_ and *p*_*e*_ give, respectively, the number of *γ* and electrons emitted per disintegration. *E*_*γ*_ is the energy of the main *γ* emission. *γ*-energies and emission probabilities for ^177^Lu, ^188^Re, ^124^I, and ^131^I were obtained from the website http://laraweb.free.fr/. Positron emissions for ^124^I and their specific abundance were taken from reference [30]. *E*_*β*_ represents the mean energy of *β*-particles emitted per disintegration. The *β*-spectra for the four different radionuclides were taken from the database on the RADAR site (http://www.doseinfo-radar.com/RADARDecay.html, October 2013). Finally, *R*_*β*_ gives the mean *β*-particle range in soft tissues.

While ^90^Y is a pure *β*-emitter, the four other radionuclides emit photons which can also interact with the biological matter inside the tumour to give birth to secondary electrons or *γ* with smaller energies. The MCNPX code is capable of studying the electron transport through matter by taking into account the loss of energy, multiple scattering angles, and “bremsstrahlung.” All these physical processes are considered by using the photon-electron mode and the default PHYS cards for electron and photons. When the different types of radiations emitted per disintegration are taken into account, the total absorbed dose to medium was determined by formula (1) [31]:

(1) Dr=21.34×Etotr×1ρ×Aλeff;Etot=∑iEir×pi,

where *E*_tot_(*r*) is the total deposited energy per unit of volume and per disintegration for the different distances “*r*” from the tumour centre determined by using the SMESH tally from MCNPX. This tally builds virtual concentric spherical shells superimposed on the geometry of the tumour surrounded by healthy lung tissues and defines the average energy, in MeV/cm³, and per emitted particle (electron or gamma) deposited into each shell located at different distances “*r*” from the tumour centre [14, 15]. The shell thickness chosen for our simulations is 0.05 cm. For ^188^Re, ^131^I, ^124^I, and ^177^Lu, *E*_tot_(*r*) was calculated by adding deposited energies, *E*_*i*_(*r*), for *γ* and *β* decays. The number of particle histories (NPS) was chosen to obtain an energy deposition per shell volume with a statistical uncertainty below 5% (1 SD). All physical processes were taken into account by choosing the photon-electron mode (MODE P E) and the default PHYS cards with a lower cut-off value for electrons and photons at 0.005 MeV. *p*_*i*_ are the total number of particles (electrons or gammas) emitted per disintegration. Values of *p*_*i*_ for *β* and *γ* decays and for the different radionuclides are given in Table 1. The total energy deposited in each spherical shell must still be divided by the density (*ρ*, in g/cm³) of the materials used to model the tumour or its surrounding healthy tissue to obtain the average energy per unit of mass and per emitted particle. *λ*_eff_ is the effective decay constant, assuming a monoexponential decay. This value is calculated by adding the physical decay constant (*λ*_phys_ = ln(2)/*T*_1/2_^phys^) and the biological clearance rate constant (*λ*_bio_ = ln(2)/*T*_1/2_^biol^). While the physical half-life is well established (Table 1), the biological half-life of the radiolabelled antibodies is more difficult to determine (Table 2). The ratio A/*λ*_eff_ represents the cumulated activity by assuming an instantaneous antibody uptake and clearance at a specific biological half-life *T*_bio_. The total activity *A* inside the tumours and surrounding healthy tissues is calculated according to the following expression:

(2) $$\begin{array}{c} A=\lambda_{phys}\times n_{u}\times n_{a}\times n_{mAb}, \end{array}$$

where *n*_*u*_ represents the total number of cell clusters. *n*_mAb_ corresponds to the maximal number of radiolabelled monoclonal antibodies capable of binding each cell cluster. Its value is determined by multiplying the surface of the cell unit by a mean 10^9^ mAb/cm² covering fraction (surface antigen concentration). The latter is defined as the number of bound antibodies per tumour surface unit and its value usually varies between 10^8^ and 10^10^ mAb/cm² [32]. Finally, *n*_*a*_ is the number of radioactive atoms per NO if direct uptake is assumed. *n*_*a*_ equals 1 if one NO contains one single radioactive atom. Note, however, that *n*_*a*_ in each NO could be reduced by applying the exponential radioactive decay law if we take into account that several days (about 2 days) are usually required for antibody maximum accumulation within the tumour [33].

To investigate how TCP and NTCP distributions evolve with an increasing number of radionuclide contained in each NO, *D*(*r*) is first converted into the biologically effective dose BED(*r*) according to formula (3) previously published and based on the linear-quadratic model [15]:

(3) $$\begin{array}{c} BED\left(r\right)=D\left(r\right)+\frac{\lambda_{eff}}{\left(\lambda_{eff}+\mu \right)}\frac{D{\left(r\right)}^{2}}{\alpha /\beta }, \end{array}$$

where *μ*, in h^−1^, is the exponential rate constant quantifying the rate of sublethal damage repair [34]. *α* and *β*, expressed in Gy^−1^ and Gy^−2^, respectively, are cellular radiosensitivities determined from cell survival curves. All biological parameters required to determine TCP and NTCP are listed in Table 2. They are assumed uniform throughout the NSCLC tumour and the lung healthy tissues for our calculations.

TCP, defined as the probability that no tumour cell cluster inside the NSCLC tumour survives irradiation, is obtained by multiplying all the shell control probability values (SCP(*r*)) from the tumour centre (*r* = 0) to the tumour surface (*r* = *r*_*T*_)

(4) $$\begin{array}{c} TCP=\overset{r_{T}}{\underset{r=0}{\prod }}SCP\left(r\right)=\overset{r_{T}}{\underset{r=0}{\prod }}{\left(p_{TCC}\left(r\right)\right)}^{K_{r}}, \end{array}$$

where SCP(*r*) gives the probability that no tumour cell cluster inside each spherical mesh located at a distance *r* from the tumour centre survives irradiation. *K*_*r*_ is the number of TCC within the concentric spherical shell located at a distance “*r*” from the tumour centre and *P*_TCC_ is the probability to kill a TCC determined by the Poisson expression:

(5) $$\begin{array}{c} p_{TCC}\left(r\right)=exp\left\{-N_{Cells}\cdot exp\left\{-\alpha_{TCC}\cdot BED\left(r\right)\right\}\right\}, \end{array}$$

where *α* is the tumour cell radiosensitivity and *N*_Cells_ is the number of tumour cells inside each TCC before irradiation obtained by multiplying the density *ρ* from Table 2 with the TCC volume.

To assess the risk of pneumonitis after RIT treatment, TCP curves will be compared to three different NTCP models. The first one is the phenomenological Lyman-Kutcher-Burman (LKB) model expressed by

(6) $$\begin{array}{c} NTCP_{LKB}=\frac{1}{\sqrt{2\pi }}\overset{t}{\underset{-\infty }{\int }}e^{-x^{2}/2}dx\;with\;\;t=\frac{MLD-TD_{50}}{m\cdot TD_{50}}, \end{array}$$

where TD_50_, in Gy, represents the dose for a 50% complication rate, *m* controls the NTCP curves slope, and MLD gives the mean lung dose deposited into the pair of lungs. TD_50_ and *m* have never been determined for RIT. So we chose to use the values given by Seppenwoolde et al. for external radiotherapy, that is, a TD_50_ = 30.8 Gy and *m* = 0.37 [35]. For the MLD, we assumed that the dose deposited into a 4.0 cm thick lung shell corresponded to the MLD deposited in both lungs.

The second NTCP model used in this paper for describing the risk of pneumonitis a few months after treatment is the relative seriality model (SER), initially proposed by Källman et al. [36, 37]

(7) $$\begin{array}{c} NTCP_{SER}={\left[1-{\left(1-p_{FSU}^{s}\right)}^{K_{Lg}}\right]}^{1/s}, \end{array}$$

where “*s*” is the degree of seriality for the lung organ. Usually, its value is close to zero with *s* = 0.0061. *K*_Lg_ equals 76 394 192 and represents the total number of FSUs in the whole lung. This value was obtained by dividing a mean lung volume of 5000 cm³ by the volume of one FSU. *p*_FSU_ is the probability of damaging one FSU, calculated with a similar expression to (5), except that *N*_Cell_ is the number of cells in one FSU before its irradiation and *α* is the lung tissue radiosensitivity. *N*_Cell_ and *α* are given in Table 2. BED(*r*) in expression (5) will also be replaced by a mean value of the BED within a 4.0 cm thick lung spherical shell.

Finally, TCP curves will also be compared to a third NTCP model for which a minimum number of FSUs (also called tissue rescue units, TRUs) exist and maintain the whole organ function. So, in this last model, we assume that complication rises when all TRUs within the lungs are killed:

(8) NTCPTRU =∏r>rTrLgexp−NTRUr·exp−αFSU·BEDr·vc,

where *N*_*r*_ gives the initial number of TRU in each spherical shell located at a distance “*r*” from the tumor center and *α* describes FSU radiosensitivity; BED(*r*) is the biological effective dose at a distance “*r*” from the tumour centre; *c* reflects the volume effects; *v* is the fractional partial volume defined by the ratio *N*_tot_/*N*_Lg_ (*N*_tot_ is the total number of TRUs and *N*_Lg_ is the number of TRUs within the pair of lungs); *N*_*r*_ is the number of TRUs at a distance “*r*” from the tumour centre; *r*_Lg_ is the distance from the tumor center of the last spherical lung tumor shell, and *c* = −0.98.

## 3. Results and Discussions

### 3.1. **D**(**r**) and SCP(**r**) Calculations When One Radionuclide Is Labelled to Each Antibody

In this section, absorbed doses to medium, *D*(*r*), and shell control probability, SCP(*r*), as functions of the distance “*r*” from the tumour centre have been compared for the five different radionuclides: ^90^Y, ^177^Lu, ^131^I, ^124^I, and ^188^Re. We assume here that only one radionuclide is coupled to each antibody and that the five radionuclides are separately conjugated to antibodies with similar biological properties, such as binding affinity, covering fraction, and biological half-life. Results will be investigated to treat two small NSCLC tumour sizes (0.5 and 1.0 cm radii) filled with uniform, linear, or exponential antibody distribution. Rapid and slow antibody clearances from the tumour are taken into consideration by assuming two different biological half-lives of 2 and 6 days. Tables 3 and 4 give the values of the total doses (*D*_Tot_), in Gy, absorbed by the 0.5 and 1.0 cm radii tumours, respectively. These total absorbed doses were obtained according to formula (1) for which the deposited energy *E*_*i*_(*r*) was calculated with the MCNPX software by considering a virtual sphere with a diameter equivalent to the tumour size. Deposited doses in the centre (*D*_Cent_) and at the surface (*D*_Surf_) of the NSCLC tumour were also presented in these tables. *D*_Lung_ represents the average dose deposited in the 4.0 cm thick lung tissue shell around the tumour. This value enables treatment impact assessment on the healthy lung FSUs surrounding the tumour. By extension, we assume that it is the average dose deposited in the whole organ. This assumption probably overestimates the absorbed doses in lungs because the highest doses in RIT are mainly localized in the tumour vicinity. Finally, the three first columns of Tables 3 and 4 specify the type of radionuclide (Rad.), the shape of antibody distribution (Dist.), and the activities (*A*), in kBq, calculated according to formula (2) with a mean covering fraction of 10^9^ mAbs/cm².

#### 3.1.1. For 0.5 cm Radius Tumours

**Figure 2:**
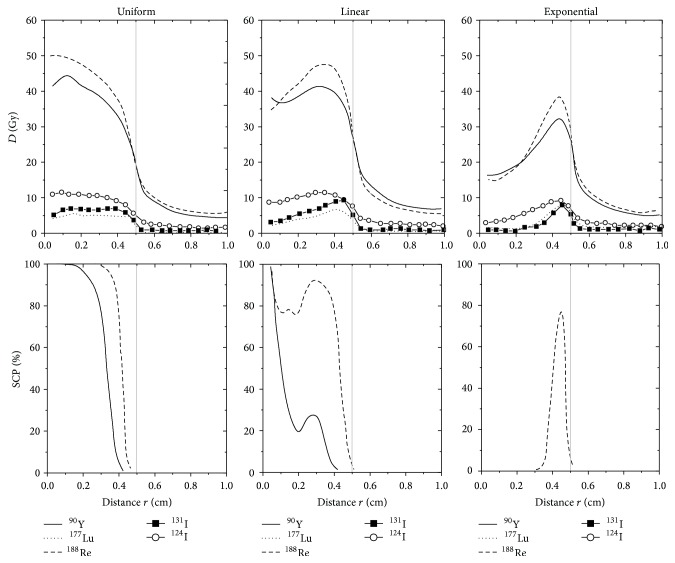
Comparison of *D*(*r*) and SCP(*r*) profiles as a function of the distance “*r*” from the tumour centre when single ^90^Y, ^177^Lu, ^188^Re, ^131^I, and ^124^I are coupled with antibodies distributed uniformly, linearly, and exponentially throughout a 0.5 cm radius tumour. The biological half-life of antibodies within the tumour corresponds to 6 days.

Doses deposited at different distances “*r*” from the tumour centre for the five different radionuclides and for the three possible antibody distributions are represented in Figure 2 when the biological half-life of the antibodies is set to 6 days. The highest values for deposited doses are found in the tumour centre when the antibody distribution is uniform, in the tumour middle when the antibodies are distributed linearly, and near the intratumoural surface for the exponential antibody distribution. Furthermore, due to the crossfire effect, the deposited doses inside the tumour for a uniform distribution of ^90^Y- and ^188^Re-labelled antibodies increase from the surface to the tumour centre. Inversely, the plateau and the sharpness of the falloff near the tumour surface for the absorbed dose of ^177^Lu or ^131^I are due to shorter emission ranges in lung tissues. As shown in Table 3, larger doses are deposited inside the tumour for the five radionuclides when the time lapse before the radiolabelled antibodies clearance increases.

The lowest values of the deposited doses inside the tumour are located at the tumour surface or in the tumour centre, depending on the radionuclide and the mAb distribution. If we look at Table 3, *D*_Surf_ is lower than *D*_Cent_ when the five different radionuclides are distributed uniformly throughout the tumour, meaning that the minimum deposited doses are found at the tumour surface. Inversely, *D*_Cent_ is lower than *D*_Surf_ when the radiolabelled antibodies are distributed exponentially within the tumour. The situation is more complicated for linear mAb distribution. Indeed, the minimum deposited doses are localized at the tumour surface for high-energy *β*-emitters (^90^Y, ^188^Re, and ^134^I) and in the tumour centre for low-energy *β*-emitters (^131^I and ^177^Lu).

Whereas strong variations of *D*_Cent_ and *D*_Surf_ appear between the three different antibody distributions, *D*_tot_ seems to remain constant for each radionuclide type. Consequently, values of *D*_tot_ strongly depend on the radionuclide choice and not on the antibody distribution type. The largest *D*_Tot_ is given for ^188^Re-labelled antibodies. However, it does not exceed 35 Gy in the best conditions of treatment, that is, a long biological half-life of 6 days and a uniform antibody distribution throughout the tumour. Such a dose clearly remains insufficient to treat 0.5 cm radius NSCLC tumours.

Figure 2 also shows values of SCP plotted against “*r*” for the five different radionuclides. These curves give a representation of cell cluster-killing efficacy at different distances “*r*” from the tumour centre and give an idea about NSCLC treatment output. For all types of antibody distributions, SCP values for ^177^Lu, ^124^I, or ^131^I at different distances “*r*” from the tumour centre remain null, indicating that these three radionuclides are not adequate to treat this solid and vascularized 0.5 cm radius tumour. By looking at the SCP curves of ^188^Re and ^90^Y from Figure 2, we also see that the spatial distribution of the antibodies plays an important role in the treatment outcome. For biological half-lives of 2 or 6 days, *D*_tot_ for uniform, linear, and exponential distribution of ^188^Re-labelled antibodies is almost the same whereas the shape of SCP curves varies strongly according to the type of antibody distribution. A similar conclusion can be drawn for ^90^Y-labelled antibodies. For both radionuclides, only a small difference of about 3 Gy is calculated between the total doses deposited by the radiolabelled antibodies distributed uniformly and exponentially, although strong differences appear in the shape of SCP curves. The slight decrease of 5 Gy when antibody distribution becomes more heterogeneous is explained by the fact that a part of the radiation is transferred from the tumour surface into healthy tissues by crossfire effects. However, with values lower than 3 Gy for the different radionuclides (cf. Table 3), doses deposited in healthy tissues surrounding the tumour remain largely below the tolerated mean radiation dose of 20 Gy for the lung [35, 45].

#### 3.1.2. For 1.0 cm Radius Tumours

**Figure 3:**
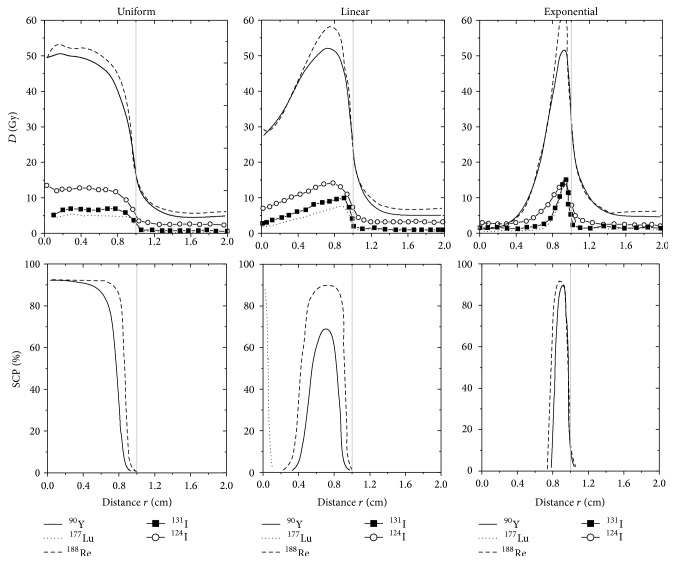
Comparison of *D*(*r*) and SCP(*r*) profiles as a function of the distance "*r*" from the tumour centre when single ^90^Y, ^177^Lu, ^188^Re, ^131^I, and ^124^I are coupled with antibodies distributed uniformly, linearly, and exponentially throughout a 1.0 cm radius tumour. The biological half-life of antibodies within the tumour corresponds to 6 days.

Figure 3 presents deposited doses, in Gy, for different radial distances when the biological antibodies half-life is 6 days but this time we are studying a 1.0 cm radius tumour. As previously observed for smaller tumours of 0.5 cm radius, *D*_Surf_ and *D*_Cent_ strongly vary with the radionuclide choice and the antibody distribution type. For uniform distribution of radiolabelled antibodies, maximal and minimal deposited doses are located in the tumour centre and at the tumour surface, respectively. For linear distribution, the highest deposited doses are found between the tumour centre and the tumour surface. The lowest deposited doses appear at the tumour surface, except for the ^124^I which presents similar values for *D*_Cent_ and *D*_Surf_. Finally, deposited doses for exponential antibody distribution are lower than 1 Gy in the central part of the tumour and the highest doses are deposited near the tumour surface. These results still show that the shape of absorbed doses is strongly affected by the antibody distribution type. Inversely, for each radionuclide, very similar values for *D*_tot_ and *D*_Lung_ between uniform, linear, and exponential antibody distributions are given in Table 4. Only a small decrease of *D*_tot_ appears when the antibodies coupled with ^90^Y, ^188^Re, or ^124^I are distributed exponentially rather than uniformly or linearly. Once again, this decrease can be justified by a crossfire effect which causes a radiation loss in the surrounding healthy tissues when the antibodies are preferentially located at the tumour surface.

As shown in Table 4, the highest total deposited doses of 39 Gy and 36 Gy were calculated for ^188^Re- and ^90^Y-labelled antibodies, respectively. However, these doses were obtained according to a long biological half-life of 6 days and a good penetration to generate a uniform activity distribution inside the tumour. Note that a decrease of the antibody biological half-life within the tumor can cause a decrease of *D*_tot_ within the tumor. Higher total deposited doses are essential to eliminate the NSCLC tumour, provided that these doses remain lower than 20 Gy in the surrounding healthy tissues. For ^90^Y and ^188^Re, the largest doses deposited 4.0 cm beyond the tumour surface remain lower than 20 Gy, even with a biological half-life of 6 days and an exponential distribution of the antibodies.

As illustrated in Figure 3, SCP values for ^131^I, ^124^I, or ^177^Lu distributed uniformly, linearly, or exponentially equal 0% as previously observed for smaller 0.5 cm radius tumours. Inversely, the shape of the SCP curves for ^90^Y and ^188^Re radionuclides varies according to the antibody distribution type. For a *T*_bio_ of 6 days, SCP curves of ^188^Re-labelled antibodies present a plateau starting from the tumour centre and stopping before the tumour surface for uniform distribution, a large peak in the middle of the tumour for linear distribution, and a narrow peak near the tumour surface for exponential distribution. Smaller and narrower SCP peaks appear for the ^90^Y-labelled antibodies. This result suggests that ^188^Re-radiolabelled antibodies have an increased chance of killing NSCLC tumours, regardless of the mAbs distributions.

Consequently, when only one radionuclide is labelled to each antibody, the highest tumour-to-lung deposited dose ratios for both tumour sizes are achieved for low-energy *β*-emitter ^177^Lu. However, the NSCLC treatment is favoured by using high-energy *β*-emitters (^90^Y and, in particular, ^188^Re) labelled to antibodies with a slow clearance (*T*_bio_ = 6 days) and a good penetration (uniform distributions) within the tumour.

### 3.2. TCP Results for Nanoobjects Containing More Than One Radionuclide

Even with a covering fraction of 10^9^ mAbs/cm², a long biological half-life of 6 days, and a good intratumoural penetration of the radiolabelled antibodies, results from Section 3.1 show that activity remains insufficient to obtain total absorbed doses up to 60–70 Gy. The logical consequence is that all TCP values remain null for both 0.5 cm and 1.0 cm radii tumours, regardless of the radionuclide used for RIT treatment or the antibodies biokinetics and spatial distribution. However, deposited doses inside the tumour could be increased by synthesizing nanoobjects containing more than one radionuclide per NO. In this section, the impact on the therapeutic effectiveness will be studied when the previously analysed radionuclides coupled to each antibody are replaced by NO containing more than one radioactive atom of ^90^Y, ^177^Lu, ^131^I, ^124^I, and ^188^Re. For this purpose, the tumour control probability (TCP) versus the number of radioactive atoms per NO will be analysed. Its value represents the probability that a tumour will be locally controlled for a given dose of radiation. A TCP value of 0% implies that doses deposited inside the tumour remain insufficient, while a TCP value of 100% predicts that the tumour will be eradicated. Our TCP curves will then be compared to NTCP values. Indeed, the latter limits our ability to administrate the dose necessary to control the tumour.

Results reported in Tables 5 and 6 investigate how the tumour size (0.5 and 1.0 cm radii) and the spatial distribution of antibodies modify the number of radioactive atoms per NO required for a maximum TCP of 100%. Both tables give values of the total doses absorbed by the tumour (*D*_Tot_, in Gy), doses in the tumour centre (*D*_Cent_, in Gy), and doses deposited at the tumour surface (*D*_surf_, in Gy), required to have a TCP value of 100%. An increase in the deposited doses inside tumours is often associated with an increase in toxicity for healthy tissues surrounding the tumour. To assess the negative effects these nanoobjects could have on the surrounding healthy tissues in 4.0 cm thick shells when TCP has reached 100%, the average doses deposited beyond the tumour surface (*D*_Lung_) are calculated for each radionuclide. To avoid lung diseases like pneumonitis, it is preferable that their values do not exceed the mean radiation dose of 20 Gy usually tolerated by healthy lung tissues. The fourth and fifth columns in Tables 5 and 6 give the number of radionuclides per antibody required to obtain a TCP of 100%. These numbers are directly scaled with a 10^9^ mAbs/cm² covering fraction and an antibody biological half-life within the tumor of 6 days: a less specific antibody with a covering fraction of 10^8^ mAb/cm² would require the number of radioactive atoms to be multiplied by ten to obtain the same TCP. The fourth and fifth columns indicate, respectively, the number of radionuclides when the uptake is supposed to be instantaneous (direct uptake) and the number of radionuclides when 2 days are required to obtain the maximum accumulation of radiolabelled antibodies within the tumour after intravenous administration (2 days uptake) [33]. For the treatment, it is evident that the more radioactive atoms per NO are required, the more difficult the synthesis of small size nanoparticles is. Finally, the three first columns of these tables, respectively, specified the type of radionuclide contained in the radioactive nanoobjects (Rad.), the type of the antibody distribution (Dist), and the tumour activity (*A*) required to achieve a TCP of 100%.

#### 3.2.1. For 0.5 cm Radius Tumours

*D*_*tot*_ *for a TCP of 100%*. For ^90^Y, ^188^Re, and ^124^I, a total deposited dose lower than 110 Gy inside the whole tumour is sufficient to achieve a TCP of 100%. This value further decreases when the antibodies are distributed linearly and uniformly throughout the tumour until about 65 and 70 Gy. With ^177^Lu- or ^131^I-nanoobjects, doses required to completely kill the tumour are higher for the three types of distribution. The total deposited doses required for a TCP of 100% remain unchanged when the biological half-life increases or decreases but the number of radioactive atoms per NO is different. The smaller the biological half-life is, the higher the required number of radioactive atoms per NO is.

Independently of antibody distribution, ^188^Re and ^90^Y require a lower number of radioactive atoms per NO to reach a maximal TCP of 100%. Note that if a direct uptake was assumed, the number of radionuclides per NO requested for a TCP of 100% would be slightly larger for ^188^Re than for ^90^Y despite a lower mean energy of *β*-particles emitted by the ^188^Re. The situation is quite different if we consider that 2 days are required to obtain a maximum accumulation of radiolabelled antibodies within the tumour (column 5 in Table 5). In this case, the number of ^188^Re per NO is higher than the number of ^90^Y per NO due to the shorter physical half-life of ^188^Re. Although the radionuclide ^124^I emits high-energy positrons, the number of atoms per NO required to achieve a TCP of 100% is larger compared to ^90^Y or ^188^Re. This increase of atoms per NO can be explained by a lower number of *β*-particles emitted per disintegration (see Table 1). Finally, ^177^Lu and ^131^I emit low-energy *β*-particles with a range lower than the cell cluster diameter used for our tumour model, meaning that the crossfire effects should be limited. Consequently, the number of radionuclides per NO requested to reach a maximal TCP value of 100% drastically increases. For all radionuclides, the exponential antibody distribution requires a larger number of radioactive atoms per NO for similar treatment efficacy.

*D*_*Surf*_ *and D*_*Cent*_ *for a TCP of 100%*. For a NSCLC tumour of 0.5 cm radius, we have previously shown that the lowest deposited dose values inside the tumour are located at the tumour surface or in the tumour centre, depending on antibody distribution, biological half-life, and the radionuclide used for the treatment. As illustrated in columns 7 and 8 of Table 6, the minimum deposited doses for a TCP of 100% range from 46 Gy to 64 Gy. These results show that a minimum deposited dose of about 55 Gy everywhere inside the tumour is required to obtain a good NSCLC treatment outcome.

*D*_*Lung*_ *for a TCP of 100%*. Treatment efficacy depends not solely on the dose absorbed by the tumour but also on the doses deposited by the nanoobjects in the surrounding healthy tissues. As observed in Table 6, average doses deposited 4.0 cm beyond the tumour surface are lower than 13 Gy for the five different radionuclides distributed uniformly and linearly through the tumour, meaning that the nanoobjects should not have too many direct negative effects on the healthy tissues surrounding the tumour. Larger deposited doses in surrounding healthy tissues are calculated for the exponential distribution of ^131^I-, ^124^I-, and ^177^Lu-nanoobjects. This result is explained by the high number of radionuclide per NO required for TCP = 100%.

Furthermore, the tumour-to-lung deposited dose ratios (*D*_Tot_/*D*_Lung_) calculated for the five different radionuclides and the three antibody distributions are similar to those previously determined for a tumour when only one radionuclide is coupled to each antibody. So, the largest values for the *D*_Tot_/*D*_Lung_ ratios are obtained for ^177^Lu-NPs, which indicates that the treatment with ^177^Lu-NOs maximizes the radiation dose to the tumour while minimizing normal tissues irradiation. However, as the number of radioactive atoms per NO is high and as ^177^Lu has a longer physical half-life compared to ^90^Y and ^188^Re, it is very important to avoid high concentrations of these ^177^Lu-nanoobjects accumulating in the nontargeted normal organs. Indeed, one isolated NO containing hundreds of atoms of ^177^Lu is harmless for the patient but an accumulation of these nanoobjects in healthy radiosensitive organs such as the red marrow may be very dangerous.

**Figure 4:**
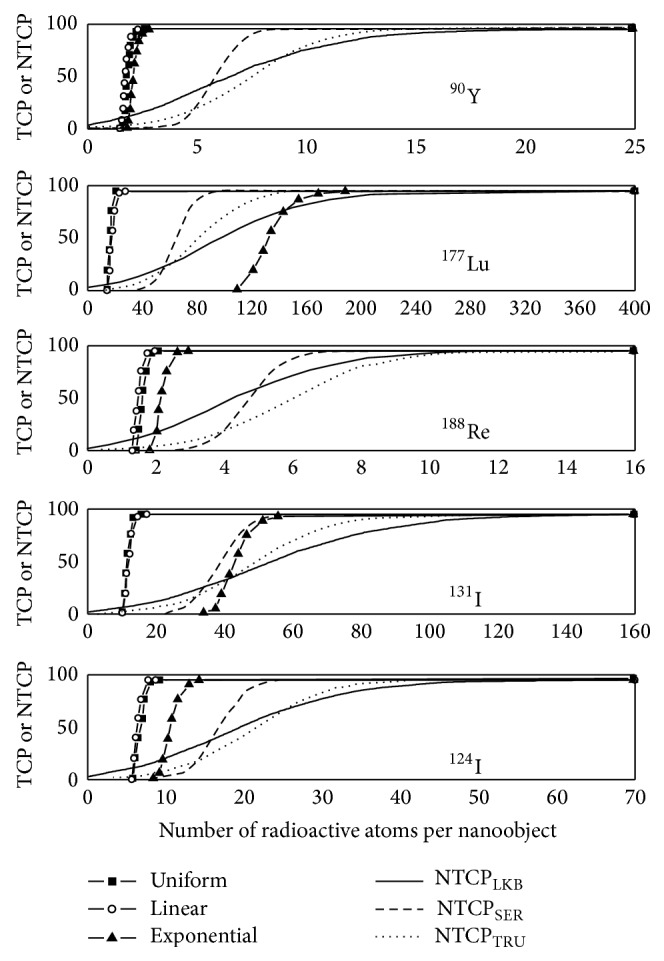
TCP values for a 0.5 cm radius tumour, calculated in %, as a function of the number of radioactive atoms included in each NO when the covering fraction corresponds to 10^10^ mAbs/cm² and *T*_bio_ = 6 days. TCP curves are represented for ^90^Y-NOs, ^177^Lu-NOs, ^188^Re-NOs, ^131^I-NOs, and ^124^I-NOs distributed uniformly, linearly, and exponentially throughout the tumour. For each graph, TCP curves are compared to NTCP curves to predict lung complications risks.

*TCP Curves*.  Figure 4 shows TCP curves, plotted for 0.5 cm radius NSCLC, in relation to the number of radioactive atoms contained in each NO when a direct uptake is considered. TCP curves are represented for the five radionuclides ^90^Y, ^177^Lu, ^188^Re, ^131^I, and ^124^I. For each radionuclide, the 3 types of antibody distributions (uniform, linear, and exponential) and for a biological half-life of 6 days were considered. TCP curves differ according to the radionuclide used for RIT and the type of antibody distribution, showing that treatment efficacy depends on the choice of *β*-emitters but also on the localization of the activity inside the tumour. NTCP curves were added to assess the risk of pneumonitis for RIT treatments. As illustrated in Figure 4, TCP curves which appear the farthest to the left on each graph are those for which the antibodies are distributed linearly or uniformly through the tumour. Small differences appear between these 2 distributions. When the nanoobjects are distributed exponentially throughout the tumour, the TCP curves shift to a higher number of radioactive atoms per NO. However, for ^90^Y, ^188^Re, and ^124^I, a maximum TCP of 100% could still be reached without too many risks of pneumonitis. Inversely, for the two low-energy *β*-emitters ^131^I and ^177^Lu, it is impossible to apply an effective treatment without causing strong lung complications. Indeed, TCP curves for both these radionuclides in Figure 4 are predominantly located to the right of the NTCP curves and tumour control without a high incidence of complications is unlikely. Note that TCP curves shift to the right when the biological half-life of the antibodies decreases, allowing similar treatment efficacy with a higher number of radionuclides per NO. The shift to the right is even more pronounced when radionuclides have a greater physical half-life such as ^177^Lu and ^131^I. Consequently, for ^124^I-nanoobjects distributed exponentially within the tumour, TCP and NTCP curves become too close to one another to insure safe treatment if the antibody biological half-life within the tumor decreases.

#### 3.2.2. For 1.0 cm Radius Tumours

*D*_*tot*_ *for a TCP of 100%*. By comparing Table 6 with Table 5, the total deposited doses to reach a maximum TCP of 100% are clearly higher when the tumour size increases. For uniform and linear antibody distributions, the total deposited doses for ^124^I, ^188^Re, and ^90^Y range from 86 and 106 Gy for a biological half-life of 6 days. Due to the low-energy of *β* emitted by ^131^I, a minimum *D*_tot_ of 135 Gy is required to obtain a TCP of 100%. Such values can be explained by the lack of crossfire effect which implies a higher difficulty to kill all the cell clusters located at the tumour surface. With values larger than 180 Gy, the total deposited dose to achieve a TCP of 100% when antibodies are distributed uniformly or linearly throughout the tumour still increases for ^177^Lu, although this radionuclide has the same mean *β*-particle range in soft tissues as ^131^I. When the antibodies are distributed exponentially throughout the tumour, the deposited doses required for a TCP of 100% greatly increase, particularly for ^90^Y, ^188^Re, and ^177^Lu which require a *D*_tot_ up to, respectively, 1320 Gy, 4220 Gy, and 3430 Gy. As previously seen in Section 3.1, the minimum deposited dose for ^90^Y, ^131^I, ^124^I, ^188^Re, and ^177^Lu was located in the tumour centre when antibodies are distributed exponentially throughout the 1.0 cm radius tumour. For the five radionuclides, doses in the tumour centre range from 52 to 56 Gy, meaning that a minimum dose of about 55 Gy is required everywhere inside the tumour to achieve a TCP of 100%. So, the important increase of *D*_tot_ for the three radionuclides ^90^Y, ^188^Re, and ^177^Lu is mainly explained by the great difficulty to irradiate all the cell clusters located in the tumour centre at about 55 Gy. Smaller increases of *D*_tot_ are observed for ^124^I and ^131^I. This probably results from the higher contribution of *γ* emissions in the total deposited energy. Indeed, *γ*-rays have a larger range in soft tissues compared to *β*-particles and favour dose depositions at a greater distance from the radionuclide. In this way, all the cell clusters located in the tumour centre, as well as those located at the tumour surface, are easier to irradiate when the ^124^I- or ^131^I-nanoobjects are exponentially distributed within the tumour.

For uniform and linear distributions of high-energy *β*-emitters (^90^Y, ^188^Re, and ^124^I), the number of radionuclides per NO does not really vary compared to smaller tumours of 0.5 cm radius. The larger activities observed in Table 6 compared to Table 5 are essentially due to a larger number of cell clusters around which the antibodies can be fixed. The number of radionuclides needed to achieve a TCP of 100% drastically increases when the ^90^Y- and ^188^Re-nanoobjects are coupled to antibodies distributed exponentially throughout the tumour. The strong activity increase calculated for exponential antibody distribution comes from a larger number of cell clusters within the tumour but also from a larger number of radioactive atoms per NO. Such a result could be explained by a great difficulty to irradiate all the cell clusters localized in the tumour centre. Consequently, the exponential distribution of both ^188^Re- and ^90^Y-nanoobjects represents a disadvantage in the treatment of solid tumours with radii equal to or larger than 1.0 cm. The latter problem is even more striking when the low-energy *β*-emitter ^177^Lu is used for internal radiation. Independently of the antibody distribution type, the larger number of radionuclides per NO observed when the tumour size increases could be explained by a great difficulty to kill all the cell clusters located at the tumour surface. A problem for irradiating cell clusters located in the tumour centre also appears when antibodies are distributed exponentially. Compared with ^177^Lu, the second low-energy *β*-emitter ^131^I seems to be less affected by this increase in the tumour size. Indeed, the shift towards larger values for the number of radionuclides per NO is smaller for ^131^I than for ^177^Lu. When the antibody distribution becomes exponential, this number even appears lower than that of ^188^Re. Such results are explained by a greater contribution of gamma rays for ^131^I and show that, for our MCNPX simulations, it is really important to consider the total (*β* and *γ*) energy deposition to determine TCP values. A similar conclusion is reached for the radionuclide ^124^I for which the lowest number of radioactive atoms per NO required to get a good treatment output is calculated when the antibody distribution is exponential. 

*D*_*Surf*_ *and D*_*Cent*_ *for a TCP of 100%*. For the three types of antibody distributions, the minimum doses deposited in the tumour centre or at the tumour surface are quite similar to the results calculated for a 0.5 cm radius tumour. Their values ranged from 51 Gy to 57 Gy, meaning that, once again, a minimum value of 55 Gy everywhere inside the tumour is required to treat NSCLC correctly. To preserve such a dose in the tumour centre when antibodies are distributed exponentially throughout the 1.0 cm radius tumour, *D*_Surf_ drastically increases to doses larger than 1450 Gy and 3800 Gy for high-energy *β*-emitters such as ^90^Y and ^188^Re, respectively. For iodine radionuclides, *D*_Surf_ decreases to lower values such as 240 Gy for ^124^I and 440 Gy for ^131^I.

*D*_*Lung*_ *for a TCP of 100%*. For the 5 different radionuclides, the deposited doses in the surrounding lung tissues are higher than the values calculated for a 0.5 cm radius NSCLC tumour, independently of antibody distribution. However, for uniform and linear antibody distributions, *D*_Lung_ remains lower than 20 Gy. When the antibodies are distributed exponentially throughout a 1.0 cm radius NSCLC tumour, the doses deposited in lung tissues are too high and NSCLC tumours cannot be treated by a single injection of radiolabelled antibodies. The best result is obtained for the ^124^I for which doses in lung tissues reached 35 Gy for a 6-day biological half-life. The surrounding healthy tissues are less affected by the radiation when the radioisotopes ^177^Lu, ^124^I, or ^131^I are used despite a large amount of radioactive atoms per NO required for a TCP of 100%. However, the synthesis of nanoobjects able to assemble such an important number of radioactive atoms remains very difficult experimentally. Furthermore, this high number of radionuclides, mixed with other nonradioactive organic or inorganic atoms and surrounded by additional macromolecules, could form nanoobjects too large to prevent uptake by the reticuloendothelial system and to promote their diffusion throughout the target tissue. This problem would be even more important if the covering fraction is decreased. 

**Figure 5:**
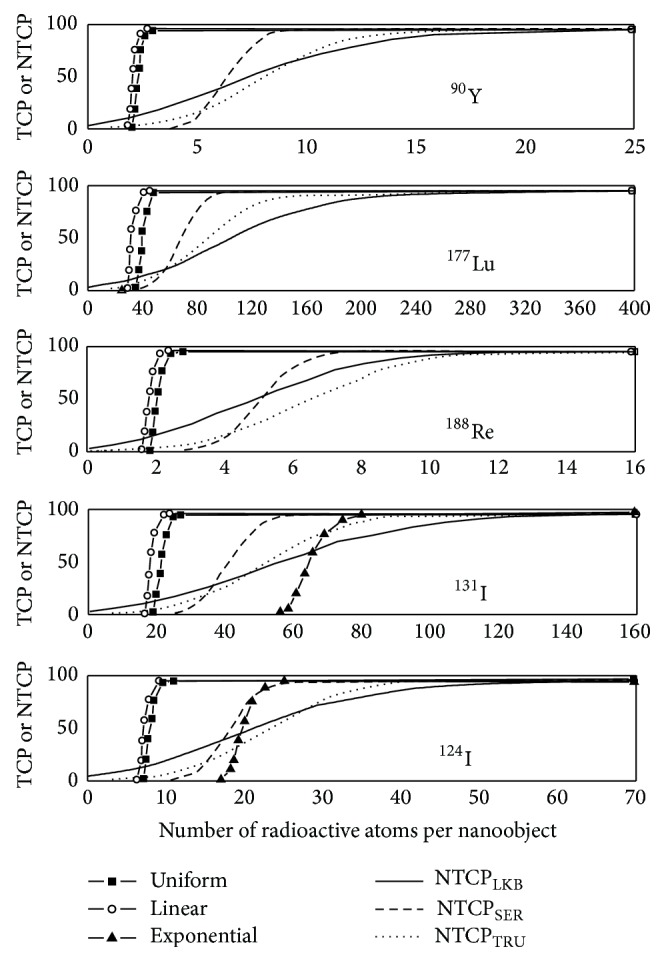
TCP values for a 1.0 cm radius tumour, calculated in %, as a function of the number of radioactive atoms included in each NO when the covering fraction corresponds to 10^10^ mAbs/cm² and *T*_bio_ = 6 days. TCP curves are represented for ^90^Y-NOs, ^177^Lu-NOs, ^188^Re-NOs, ^131^I-NOs, and ^124^I-NOs distributed uniformly, linearly, and exponentially throughout the tumour. For each graph, TCP curves are compared to NTCP curves to predict lung complications risks.

*TCP Curves*. To analyze how tumour size may influence the number of radioactive atoms per antibody required to maximize the treatment efficacy, TCP distributions have been plotted for 1.0 cm radius tumours. In Figure 5, TCP versus the number of radioactive atoms is represented for the five different radionuclides ^90^Y, ^177^Lu, ^131^I, ^124^I, and ^188^Re distributed uniformly and nonuniformly throughout the tumour. As previously stated, a biological half-life of 6 days for antibodies located inside the tumour was considered. Firstly, we can see that uniform and linear distributions of ^90^Y-, ^124^I-, or ^188^Re-nanoobjects from Figure 5 are almost similar in shape and location as those plotted in Figure 4 for smaller tumours. Consequently, tumour size does not influence treatment efficacy. Inversely, TCP curves when the ^90^Y- and ^188^Re-nanoobjects are coupled to antibodies distributed exponentially throughout a 1.0 cm radius tumour are to the right of those for complications, whereas those for smaller tumours are to the left. Note that TCP curves are localized too far away from the left of the NTCP curves to be plotted on Figure 5. Even if the shift to the right is less important for the ^124^I-NPs radiolabelled antibodies distributed exponentially throughout the tumour, maximal TCP could not be reached without lung complications.

By comparing Figure 4 with Figure 5 for both low-energy *β*-emitters ^131^I and ^177^Lu, TCP curves for uniform and linear distributions shift to the right whereas NTCP curves remain unchanged when the tumour size increases. This shift towards higher numbers of radionuclides still increases when the biological half-life decreases, meaning that the risk of pneumonitis increases.

To conclude, the tumour-to-lung deposited dose ratios for both tumour sizes are similar to those obtained when only one radioactive atom is attached to each antibody. So, the highest *D*_Tot_/*D*_Lung_ is calculated for the ^177^Lu-NO, independently of the antibody distribution or tumor size. However, after TCP and NTCP analyses, ^90^Y and ^188^Re seem the best candidates for the treatment of NSCLC tumour when antibodies are distributed uniformly or linearly. For exponential antibody distribution, the radionuclide ^124^I becomes a more adequate candidate, especially for tumours equal to or larger than 1.0 cm radius.

## 4. Conclusion

This work presents the tumour control probability analysis for solid tumours when nanoobjects (e.g., radiolabelled mAB or mAb bioconjugated nanoparticle) contain one or several *β*-emitters which could be used in radioimmunotherapy. NSCLC was selected as tumour model as its biological parameters are well reported in literature. TCP and NTCP were calculated according to tumour and normal surrounding tissue deposited doses resulting from MCNPX, a Monte Carlo electron, and photon and particles transport code. Dosimetric studies were performed for the five radioisotopes ^90^Y, ^177^Lu, ^131^I, ^124^I, and ^188^Re, for two spherical tumour sizes of 0.5 and 1.0 cm radii and for two biological half-lives. Moreover, these nanoobjects can be distributed uniformly, linearly, or exponentially throughout the tumours according to a new vascularized tumour model previously developed for MCNPX.

When nanoobjects contain only one radionuclide, simulations show that the dose absorbed by the solid tumour is insufficient for solid tumour treatment, regardless of the radionuclide used for RIT treatment or the biokinetics and spatial distributions of antibodies; even so ^188^Re and ^90^Y show the highest deposited doses within the solid tumour.

Higher total absorbed doses can easily be achieved when single radionuclides radiolabelled-mAbs are replaced by nanoobjects containing several *β*-emitters. The required number of atoms per nanoobject to get TCP = 100% changes according to numerous radionuclide physical properties such as the physical half-life, abundance of *γ* emissions, and energies of the emitted *β*-particles and *γ* particles, but also according to the biological properties of the nanoobjects such as their concentration inside the tumour, their spatial distribution, or their biological half-life. These results confirm the importance of performing individual treatment for each patient. The total absorbed doses to obtain excellent tumour control vary between 60 Gy and more than 5000 Gy depending on the radionuclide choice, tumour size, and nanoobjects spatial distribution. ^90^Y and ^188^Re are the choice if one wants to minimize the number of radioisotopes per nanoobject.

Finally, higher absorbed doses are often synonymous with higher toxicity. Consequently, NTCP usually limits our ability to administer the doses required to control the tumour. For small 0.5 cm radius tumours, simulations do not show any toxicity problems. In this case, ^90^Y and ^188^Re remain the best candidates because they require the lowest number of radionuclides per nanoobject to reach a TCP of 100%. On the other hand, for larger 1.0 cm radius tumours, toxicity to the surrounding healthy tissue is observed when the nanoobjects are distributed exponentially throughout the tumour. This toxicity increases strongly for high-energy *β*-emitters and ^124^I becomes more appropriate to cure solid tumours. Such results indicate the importance of finding new strategies to insure the penetration of nanoobjects inside tumours.

## Figures and Tables

**Table 1:** Physical characteristics of the different radionuclides studied in this work.

Nuclide	*T*_1/2_^phys^ (h)	*p*_*γ*_	*E*_*γ*_ (MeV) [abundance (%)]	*p*_*e*_	*E*_*β*_ (MeV)	*R*_*β*_ (mm)
^90^Y	64.1	—	—	1.0000	0.935	4
^177^Lu	161	0.1722	0.208 [6.1]	1.0000	0.133	0.26
^124^I	100	1.6380	0.603 [62.9]	0.2327	0.686 0.974	4.1
^131^I	192	1.0980	0.365 [85.3]	1.0000	0.132	0.26
^188^Re	16.9	0.1981	0.155 [15]	1.0000	0.764	3.1

**Table 2:** Biological parameters used for TCP calculations for NSCLC tumour and healthy lung tissue.

Biological factors	Tumour		Healthy tissues	
	Tumoral tissues	References	Healthy tissues	References
*T* ^1/2^_bio_ [d]	2–6	[21]	3	[21]
*α*/*β* [Gy]	10	[38], [34, 39, 40]	3	[38], [34, 39, 40]
*μ* [h^−1^]	1.39	[38, 41]	0.46	[34, 38, 42]
*α* [Gy^−1^]	0.35	[39]	0.031	[43]
Cellular density [#/cm³]	9.5 × 10^7^	[44]	9.5 × 10^7^	[44]

**Table 3:** Comparison of the total absorbed dose (*D*_Tot_), absorbed doses in the centre (*D*_Cent_), and absorbed doses at the surface (*D*_Surf_) or surrounding the tumour (*D*_Lung_), in Gy, when ^90^Y, ^177^Lu, ^131^I, ^124^I, and ^188^Re are separately linked to antibodies distributed uniformly, linearly, and exponentially inside a 0.5 cm radius spherical tumour. Two different biological half-lives of 2 and 6 days are considered here.

Rad.	Dist.	*A* [kBq]	*D*_Lung_ [Gy]	Absorbed dose [Gy]					
				*T*_1/2_^bio^ = 2 days			*T*_1/2_^bio^ = 6 days		
				*D*_Tot_	*D*_Cent_	*D*_Surf_	*D*_Tot_	*D*_Cent_	*D*_Surf_
^90^Y	Uni.	87.32	4.17	19.15	27.03	13.12	30.94	43.67	21.19
	Lin.		4.18	18.41	18.69	13.98	29.75	30.20	22.59
	Exp.		4.18	16.85	10.22	16.88	27.23	16.52	27.28

^131^I	Uni.	29.23	0.64	2.67	3.16	1.63	5.73	6.78	3.49
	Lin.		0.64	2.65	1.65	1.91	5.68	3.55	4.10
	Exp.		0.64	2.58	0.42	3.14	5.53	0.90	6.72

^124^I	Uni.	55.87	1.46	4.54	6.12	3.24	8.27	11.15	5.91
	Lin.		1.46	4.39	4.17	3.47	8.00	7.60	6.32
	Exp.		1.46	4.04	2.03	4.09	7.36	3.70	7.44

^177^Lu	Uni.	0.94	0.38	2.16	2.61	1.22	4.44	5.37	2.52
	Lin.		0.38	2.15	1.18	1.50	4.43	2.43	3.09
	Exp.		0.38	2.12	0.16	2.70	4.35	0.34	5.55

^188^Re	Uni.	34.78	5.22	28.87	40.32	19.15	34.90	48.74	23.15
	Lin.		5.22	27.87	27.02	20.92	33.70	32.66	25.29
	Exp.		5.23	25.49	12.90	26.05	30.82	15.60	31.49

Computed dose error is estimated to 5% (1 SD).

**Table 4:** Comparison of the total absorbed dose (*D*_Tot_), absorbed doses in the centre (*D*_Cent_), and absorbed doses at the surface (*D*_Surf_) or surrounding the tumour (*D*_Lung_), in Gy, when ^90^Y, ^177^Lu, ^131^I, ^124^I, and ^188^Re are separately linked to antibodies distributed uniformly, linearly, and exponentially inside a 1.0 cm radius spherical tumour. Two different biological half-lives of 2 and 6 days are considered here.

Rad.	Dist.	*A* [kBq]	*D*_Lung_ [Gy]	Absorbed dose [Gy]					
				*T*_1/2_^bio^ = 2 days			*T*_1/2_^bio^ = 6 days		
				*D*_Tot_	*D*_Cent_	*D*_Surf_	*D*_Tot_	*D*_Cent_	*D*_Surf_
^90^Y	Uni.	684.13	3.84	22.2	29.8	11.7	35.8	48.1	18.9
	Lin.		3.84	21.6	14.5	13.2	34.9	23.5	21.3
	Exp.		3.87	18.9	0.78	20.8	30.6	1.26	33.5

^131^I	Uni.	228.29	0.60	2.67	3.06	0.97	5.73	6.55	2.09
	Lin.		0.60	2.66	1.23	1.13	5.71	2.65	2.42
	Exp.		0.60	2.61	0.31	2.43	5.60	0.67	5.20

^124^I	Uni.	436.97	1.35	5.14	6.36	3.02	9.36	11.58	5.51
	Lin.		1.35	5.05	3.30	3.35	9.19	6.01	6.10
	Exp.		1.35	4.56	1.11	5.08	8.31	2.02	9.25

^177^Lu	Uni.	222.32	0.36	2.07	2.25	0.53	4.26	4.63	1.09
	Lin.		0.36	2.07	0.75	0.65	4.26	1.53	1.34
	Exp.		0.36	2.06	0.03	1.73	4.24	0.05	3.55

^188^Re	Uni.	2609.61	4.83	32.0	40.9	16.2	38.7	49.5	19.6
	Lin.		4.83	31.3	19.3	18.4	37.9	23.3	22.2
	Exp.		4.86	27.9	0.54	31.0	33.8	0.65	37.5

Computed dose error is estimated to 5% (1 SD).

**Table 5:** Total absorbed dose (*D*_Tot_), absorbed doses at the surface (*D*_Surf_), and absorbed doses at the centre (*D*_Cent_) or surrounding the tumour (*D*_Lung_), in Gy, required to obtain a maximum TCP of 100% when nanoobjects containing ^90^Y, ^131^I, ^124^I, ^177^Lu, and ^188^Re are coupled to antibodies having a *T*_bio_ of 6 days and distributed uniformly, linearly, and exponentially inside a 0.5 cm radius spherical tumour. *N*_NO_: number of radioactive atoms per nanoobjects required for TCP = 100%.

Rad.	Dist.	*A* [kBq]	*N*_NO_		Dose			
			Direct uptake	2 days of uptake	*D*_Tot_ [Gy]	*D*_Cent_ [Gy]	*D*_Surf_ [Gy]	*D*_Lung_ [Gy]
^90^Y	Uni.	370	2	4	77	108	52	10
	Lin.	370	2	4	69	70	52	10
	Exp.	370	3	5	79	48	79	12

^131^I	Uni.	370	15	18	88	104	54	10
	Lin.	370	17	20	95	59	68	11
	Exp.	1850	61	72	336	55	409	39

^124^I	Uni.	370	9	13	74	100	53	13
	Lin.	370	8	12	67	64	53	12
	Exp.	740	15	21	112	56	113	22

^177^Lu	Uni.	740	21	26	95	114	54	8
	Lin.	740	26	33	117	64	82	10
	Exp.	6660	188	232	820	63	1046	72

^188^Re	Uni.	740	2	15	72	101	48	11
	Lin.	740	2	14	64	62	48	10
	Exp.	1110	3	21	90	46	92	15

**Table 6:** Total absorbed dose (*D*_Tot_), absorbed doses at the surface (*D*_Surf_), and absorbed doses in the centre (*D*_Cent_) or surrounding the tumour (*D*_Lung_), in Gy, required to obtain a maximum TCP of 100% when nanoobjects containing ^90^Y, ^177^Lu, and ^188^Re are coupled with antibodies having a *T*_bio_ of 6 days and distributed uniformly, linearly, and exponentially inside a 1.0 cm radius spherical tumour. *N*_NO_: number of radioactive atoms per nanoobjects required for TCP = 100%.

Rad.	Dist.	*A* [kBq]	*N*_NO_		Dose			
			Direct uptake	2 days of uptake	*D*_Tot_ [Gy]	*D*_Cent_ [Gy]	*D*_Surf_ [Gy]	*D*_Lung_ [Gy]
^90^Y	Uni.	1850	3	5	106	142	56	11
	Lin.	1850	3	4	92	62	56	10
	Exp.	29600	43	73	1324	54	1450	167

^131^I	Uni.	6290	27	33	157	180	57	16
	Lin.	5550	24	28	135	63	57	14
	Exp.	19240	85	101	474	56	440	51

^124^I	Uni.	4440	10	14	96	119	57	14
	Lin.	4070	9	13	86	56	57	13
	Exp.	11470	26	36	216	52	240	35

^177^Lu	Uni.	14430	52	65	224	243	57	19
	Lin.	11470	43	52	182	65	57	16
	Exp.	271210	996	1224	4220	53	3539	362

^188^Re	Uni.	6660	3	19	101	129	51	13
	Lin.	5920	2	16	87	54	51	11
	Exp.	264920	102	726	3432	56	3805	494
